# High mortality in patients with *Mycobacterium avium* complex lung disease: a systematic review

**DOI:** 10.1186/s12879-018-3113-x

**Published:** 2018-05-03

**Authors:** Roland Diel, Marc Lipman, Wouter Hoefsloot

**Affiliations:** 1 LungenClinic Grosshansdorf, Wöhrendamm 80, 22927 Großhansdorf, Germany; 2 Institute for Epidemiology, University Hospital Schleswig Holstein, Campus Kiel, Kiel, Germany; 3 Division of Medicine, UCL Respiratory, University College London & Royal Free Hospital London NHS Foundation Trust, London, NW3 2QG UK; 4 Department of pulmonary diseases, Geert Grooteplein Zuid 10, 6525 GA Nijmegen, The Netherlands

**Keywords:** Infectious disease, Nontuberculous mycobacteria, NTM, Survival outcome

## Abstract

**Background:**

The incidence of nontuberculous mycobacterial (NTM) pulmonary disease caused by *Mycobacterium avium* complex (MAC) in apparently immune-competent people is increasing worldwide. We performed a systematic review of the published literature on five-year all-cause mortality in patients with MAC lung disease, and pooled the mortality rates to give an overall estimate of five-year mortality from these studies.

**Methods:**

We systematically reviewed the literature up to 1st August 2017 using PubMed® and ProQuest Dialog™ to search Medline® and Embase® databases, respectively. Eligible studies contained > 10 patients with MAC, and numerical five-year mortality data or a treatment evaluation for this patient group. Mortality data were extracted and analysed to determine a pooled estimate of all-cause mortality.

**Results:**

Fourteen of 1035 identified studies, comprising 17 data sets with data from a total of 9035 patients, were eligible. The pooled estimate of five-year all-cause mortality was 27% (95% CI 21.3–37.8%). A high degree of heterogeneity was observed (I^2^ = 96%). The mortality in the data sets varied between 10 and 48%. Studies predominantly including patients with cavitary disease or greater comorbidity reported a higher risk of death. Patients in Asian studies tended to have a lower mortality risk. Predictors of mortality consistent across studies included male sex, presence of comorbidities and advanced patient age.

**Conclusions:**

Despite high heterogeneity, most studies in patients with MAC pulmonary disease document a five-year all-cause mortality exceeding 25%, indicating poor prognosis. These findings emphasise the need for more effective management and additional prospective mortality data collection.

**Electronic supplementary material:**

The online version of this article (10.1186/s12879-018-3113-x) contains supplementary material, which is available to authorized users.

## Background

Nontuberculous mycobacteria (NTM) are ubiquitous environmental bacteria, present in soil and water sources [1]. NTM are thought of as opportunistic pathogens, with disseminated NTM disease being seen in patients with systemic impaired immunity (e.g. HIV) [2, 3]. Interest in NTM pulmonary disease (NTM-PD) is increasing due to its growing prevalence in non-HIV populations [2]. It can occur in the context of lung disease caused by, for example, bronchiectasis, chronic obstructive pulmonary disease (COPD) or cystic fibrosis (CF), and also in people with apparently normal lungs [2, 3].

NTM-PD symptoms are nonspecific and variable; patients may present with both respiratory and systemic complaints, which may relate to underlying lung disease [2]. NTM-PD usually manifests radiologically with fibrocavitary or nodular/bronchiectatic forms [2]. NTM-PD diagnosis is generally made when the American Thoracic Society/Infectious Diseases Society of America (ATS/IDSA) diagnostic criteria are met [2].

MAC is considered to be the most common cause of NTM-PD [4]. It comprises various mycobacterial species, including *M. intracellulare, M. avium* (which has four subspecies), and several other less frequently isolated species including *M. chimaera* [5, 6]. The decision to treat MAC infections depends on the patient’s health status and risk of disease progression. According to published recommendations, patients with nodular/bronchiectatic MAC disease should be offered a combination of macrolide (clarithromycin or azithromycin), rifampin or rifabutin, and ethambutol [2, 4]. In patients with fibrocavitary or severe nodular/bronchiectatic disease, addition of parenteral aminoglycosides may be considered [2]. Many are, however, refractory to first-line therapy and do not achieve sustained culture conversion [7]. Effective treatment choices for these people are few, essentially limited to intensification or modification of the first-line regimen or surgical resection of infected lung tissue [7].

MAC lung disease natural history and long-term outcomes are poorly documented, particularly at the population level [8]. A retrospective chart review of patients from Oregon, USA with respiratory NTM isolates found that the median time to death was 3.6 (range 0–7.7) years for cases meeting ATS/IDSA diagnostic criteria [2] and 3.7 (range 0.0–8.6) years for those who did not (*p* = 0.63). Here, 55% of the cases and 61% of the non-cases died during the follow-up period (2007–2014), with no statistically significant difference in five-year mortality between cases and non-cases [8].

A previous systematic review of reported treatment outcomes in patients with MAC lung disease, based on a pooled analysis of 28 studies carried out between 1977 and 2004, found overall mortality to be 17% (95% confidence interval [CI] 15–18%) [9]. However, this mainly included studies of short duration, and the calculated mortality rates did not account for different patient follow up-times within the studies [9]. Thus, it is not possible to draw firm conclusions regarding longer-term mortality from this report. Another recent systematic review sought to examine comorbidities, health-related quality of life and mortality associated with NTM disease in various patient populations [10]. Again, variable follow-up times in the included studies (30 days to over 10 years) limited the understanding of long-term mortality. Moreover, no differentiation was made between NTM-PD and NTM-non-PD, or different NTM species [10].

We therefore sought to systematically review the published literature for data on long-term mortality in patients with MAC lung disease, pool five-year mortality results to gain an estimate of overall five-year all-cause mortality in these patients, and explore study characteristics that may have contributed to variability in mortality reports or predict patient outcome.

## Methods

### Data sources

Database searches were carried out in Medline® and Embase®, using PubMed® and ProQuest Dialog™ search tools, respectively, with a cut-off of 1st August 2017, according to the Preferred Reporting Items for Systematic Reviews and Meta-Analyses (PRISMA) guidelines [11]. English language studies were selected. The search strategy applied to each database is described in the Additional file 1. Duplicates, case reports, nonclinical and animal studies were excluded, as were conference abstracts, newspaper articles, notes, news, biography, conference reviews, errata and lectures.

### Study selection

Relevant studies were independently selected by two reviewing authors (WH and RD), who screened the article title and abstract initially, and then went on to review the article full text as needed. Studies were included if they reported five-year all-cause mortality in cohorts of patients with MAC lung disease, or NTM-PD cohorts where the majority of patients (≥75%) had MAC lung disease. No restrictions were made regarding study design, patient subpopulation, or data collection (prospective or retrospective). Studies with fewer than ten patients were excluded because of uncertainty about validity of the presented data and outcome in smaller studies.

### Data extraction

The following data were extracted from the selected publications: five-year all-cause mortality, proportion of MAC-attributable deaths, factors predicting all-cause mortality, all-cause mortality in patients with fibrocavitary or nodular/bronchiectatic disease, and MAC-related mortality in patients with fibrocavitary or nodular/bronchiectatic disease.

### Statistical analysis

Heterogeneity in reported mortality rates was quantified in terms of the Q- and I^2^-statistics. The Q-statistic is based on the chi-squared test and assesses deviation between individual study effect and the pooled effect across studies. A large Q-value relative to its degree of freedom provides evidence of heterogeneity of the measured outcome (variation in outcome estimates beyond chance). The I^2^-statistic describes the percentage of the variability in outcome estimates due to heterogeneity rather than sampling error (chance). Five-year mortality rates were pooled across the studies using a random-effects model. The analysis was performed using *Review Manager (RevMan version 5.3. Copenhagen: The Nordic Cochrane Centre, The Cochrane Collaboration, 2014* software).

## Results

### Study selection and identified studies

**Fig. 1:**
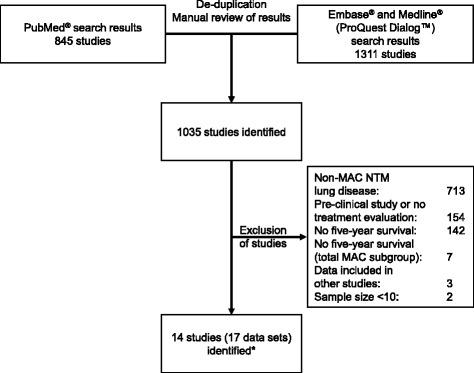
Flow chart describing the selection of studies and data sets included in the analysis.*Three of the identified publications contained data sets for two cohorts of patients, and these are considered separately here. MAC, Mycobacterium avium complex; NTM, nontuberculous mycobacterium

The PubMed® search of Medline® returned 845 studies, and the ProQuest Dialog search (using Embase® and Medline® databases) returned 1311 studies. Following comparison of the results and de-duplication, 1035 publications remained. This selection was screened by title, abstract content and full text if needed. Following the exclusion criteria stated in the Methods section, 14 studies comprising 17 data sets with data from 9035 patients remained for analysis. A flowchart depicting this selection process is shown in Fig. 1.

The identified studies and their key characteristics are listed in Table 1 [12–25]. Among these 17 data sets, nine were retrospective medical chart review studies [12–19], five were retrospective population registry analyses [20–23] and three were from prospective, randomised studies [24, 25]. Three studies included data from two cohorts of patients with MAC lung disease, and these are considered separately for the purposes of this analysis [19, 23, 25]. The number of patients with MAC in the studies ranged from 45 to 5543. Two studies examined patients with other NTM infections (with the data for the MAC subgroup considered for this analysis) [20, 21], and one investigated nodular/bronchiectatic MAC lung disease [18]. Three studies focused on newly-diagnosed MAC lung disease [13, 14, 21]. Two of the included studies covered NTM-PD, however the majority of the patients in these studies were diagnosed with MAC lung disease [22, 23].

**Table 1:** Characteristics of the identified studies

Data set	Country and year	Diagnosis	N	Age (years)	Female sex (%)	NB (%)	FC (%)	NB + FC (%)	Unknown, unclassified or other (%)	Therapy	Five-year mortality rate (%) (95% CI)
Retrospective medical chart reviews											
1	USA, 1973 [12]	MAC lung disease^b^	45	49% > 50	0	NR	81.0 (multiple cavities in 50%)	NR	11.0 (unknown)	1–3 drugs: 53% ≥4 drugs: 47% Adjunctive surgical treatment: 42% Duration: 5 years	40 (21.5–58.5)
2	Japan, 2012 [13]	Newly-diagnosed MAC lung disease^‡^	634	68.9 (mean) ± 11.4 (SD)	58.5	82.9	11.5	3.3	2.3 (unclassified)	First-line antibiotic therapy: 50.9% Duration > 3 months	23.9 (20.1–27.7)
3	Japan, 2012 [14]	Newly diagnosed MAC lung disease^‡^	78	65.2 (mean) ± 12.6 (SD)	60.3	59.0 (bronchiectatic)	26.0	NR	NR	Various treatment regimens: 69% Untreated: 31% Duration NR	25.6 (14.4–36.8)
4	Japan, 2013 [15]	Rheumatoid arthritis and MAC lung disease^‡^	82	67.6 (mean) ± 10.3 (SD)	70.7	59.8	13.4	18.3	8.5 (other)	1 or 2 drug regimens, Treatment for rheumatic disease Duration > 3 months	32.8 (20.4–45.2)
5	Japan, 2014 [16]	MAC lung disease^‡^	309	67.0 (mean) ± 13.7 (SD)	64.7	NR	NR	NR	NR	Standard 3-drug regimen including clarithromycin: 131 patients (42.4%) Duration > 6 months for 108 regimens. Pulmonary resection: 5.1%	10.0 (6.8–13.1)
6	UK, 2014 [17]	Non-cystic fibrosis bronchiectasis and coexisting MAC infection	52	63.1 ± 12.7	69.2	NR	NR	NR	NR	NR	21 (8.5–33.5)
7	Japan, 2015 [18]	Nodular/ bronchiectatic MAC lung disease, based on HRCT of the chest^‡^	782	68.1 (mean) ± 11.1 (SD)	68.5	NR	15.0	NR	NR	First line antibiotic therapy, 1–5 drug regimen: 19.6% Duration > 3 months	12.5 (10.0–15.0)
8	Japan, 2017 [19]^,a^	MAC lung disease	368	72 (mean) ± 10 (SD)	59.0	81.0	11.1	1.6	9.5	165 treated patients; Clarithromycin + ethambutol + rifampicin (79.3%); other regimens (20.7%)	23 (17.7–27.3)
9	Japan, 2017 [19]^,a^	MAC lung disease	118	70 (mean) ± 10 (SD)	55	85.6	11.9	0	2.5	66 treated patients; Clarithromycin + ethambutol + rifampicin (79.3%); other regimens (20.7%)	15 (7.8–21.6)
Retrospective population registry analyses											
10	Denmark, 2010 [20]	Prevalent NTM-PD^‡^ (MAC subgroup considered)	425	61.2 (mean) ± 16.5 (SD)	41.0	NR	NR	NR	NR	NR	39.7 (33.7–45.7)
11	Canada, 2017 [21]	MAC lung disease^‡^	5543	70 (median), IQR 50–78	53.0	NR	NR	NR	NR	NR	33.3 (31.8–34.8)
12	Japan, 2017 [22]	NTM-PD^§^	125	60 (median) IQR 49–66	66.0%	NR	NR	NR	NR	≥3 drug regimen including clarithromycin 76%; 2 drug regimen including clarithromycin 2%; clarithromycin monotherapy 4%; non-clarithromycin regimen 5%	16 (7.8–21.6)
13	USA 2017 [23]^,a^	NTM-PD (meeting ATS/IDSA criteria) treated with pulmonary resection^‖^	178	66.1 (mean) ±14.6 (SD)	60	NR	NR	NR	NR		37 (27.6–45.4)
14	USA, 2017 [23]^,a^	NTM-PD (not meeting ATS/IDSA criteria) treated with pulmonary resection^‖^	138	62.4 (mean) ±17.3 (SD)	51	NR	NR	NR	NR	NR	33 (23.7–43.0)
Prospective, randomized studies											
15	UK and Scandinavia, 2002 [24]	MAC lung disease^¶^	75	64 (mean)	46.7	NR	61	NR	NR	Rifampicin +ethambutol ±isoniazid Duration: 2 years	36.0 (22.4–49.6)
16	UK, Denmark, Sweden and Italy, 2008 [25]^,a^	MAC lung disease^¶^	83	65 (mean)	51.8	NR	69	NR	NR	Rifampicin +ethambutol +clarithromycin ±immunotherapy Duration: 2 years	48.0 (33.1–62.9)
17	UK, Denmark, Sweden and Italy, 2008 [25]^,a^	MAC lung disease^¶^	87	65 (mean)	49.4	NR	66	NR	NR	Rifampicin +ethambutol +ciprofloxacin ±immunotherapy Duration: 2 years	30.0 (18.5–41.5)

Studies are ordered within categories by year of publication

*ATS/IDSA* American Thoracic Society/Infectious Diseases Society of America, *CI* confidence interval, *FC* fibrocavitary disease, *HRCT* high resolution computed tomography, *IQR* interquartile range, *MAC Mycobacterium avium* complex, *NB* nodular/bronchiectatic disease, *NR* not reported, *NTM* nontuberculous mycobacterium, *PD* pulmonary disease, *SD* standard deviation

^a^Mortality data were provided for two differently treated cohorts of patients with MAC lung disease. ^b^Pulmonary parenchymal disease by chest radiograph, sputum or bronchial wash containing *M. intracellulare*, physician’s opinion that *M. intracellulare* caused the disease. ^‡^Disease fulfilled 2007 ATS/IDSA criteria. [2] ^§^This study included primarily patients with MAC lung disease (86%) [22]. ^‖^These data sets included primarily MAC lung disease patients (84% in full cohort, 89% of those meeting ATS/IDSA criteria [data set 13], 78% of those not meeting ATS/IDSA criteria [data set 14]) [23]. ^¶^Sputum culture positive for MAC on at least two occasions separated by at least a week, radiographic changes compatible with mycobacterial pulmonary disease, and/or clinical evidence of such disease

### Mortality rates in the identified studies

**Fig. 2:**
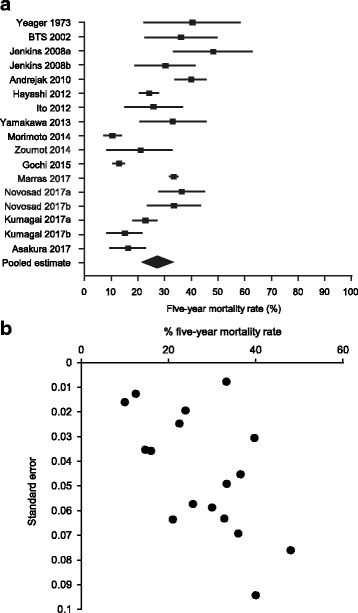
Analysis of five-year mortality in selected data sets. **a** Forest plot of five-year all-cause mortality rates in the identified data sets. Results are plotted ± 95% confidence interval (CI). **b** Funnel plot of five-year all-cause mortality versus standard error from selected data sets. BTS; The Research Committee of the British Thoracic Society

The five-year all-cause mortality data from each study, including the ranges and pooled estimate, are shown in Fig. 2a. The mortality in the studies ranged from 10.0% (95% CI 21.5–58.4%) to 48.0% (95% CI 33.1–62.9%). Pooling data from all 17 data sets using a random effects model, the overall estimate of five-year all-cause mortality was 27% (95% CI 21.3–33.0%). The I^2^ statistic was 96% and the Q-statistic was 365.1, indicating a high level of study heterogeneity. This is also demonstrated in a funnel plot of data from the selected studies (Fig. 2b).

### MAC-related and all-cause mortality

**Fig. 3:**
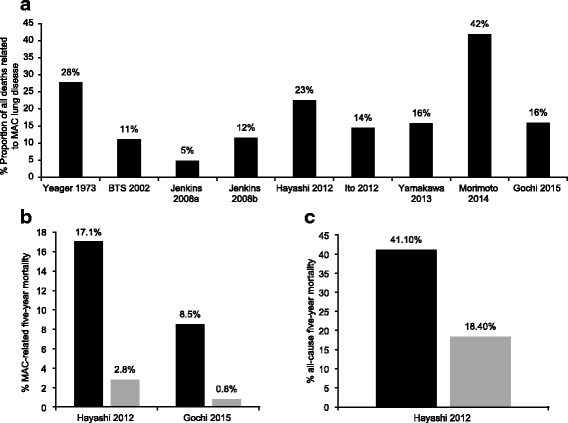
MAC-related five-year mortality and cavitary disease in selected data sets. **a** The proportion of all deaths related to MAC lung disease in the identified data sets. **b** Fibrocavitary disease and MAC-related five-year mortality. Black bars indicate fibrocavitary disease, grey bars indicate nodular/bronchiectatic disease. **c** Fibrocavitary disease and all-cause five-year mortality. Black bars indicate fibrocavitary disease, grey bars indicate nodular/bronchiectatic disease

The proportion of all MAC-attributable deaths was reported by nine studies, and these data are shown in Fig. 3a. MAC-related five-year all-cause mortality varied between 5% [25] and 42% [16]. Predictors of all-cause mortality are listed in Table 2. Several factors appeared to be consistent across studies. These include male sex [13, 16, 18–21, 24], presence of comorbidities [13–17, 19–21, 23], and advanced patient age [13, 16, 18, 20–22, 24]. Predictors of better outcome include surgical treatment [12] and nodular or bronchiectatic disease [15].

**Table 2:** Predictors of mortality in the identified studies, if any

Data set	Negative association with all-cause mortality	Positive association with all-cause mortality	Reference
1	Surgical treatment		Yeager 1973 [12]
2		Male sex Age ≥ 70 years Presence of systemic and/or respiratory comorbidity FC disease BMI < 18.5 kg/m^2^ Anaemia Hypoalbuminemia Erythrocyte sedimentation rate ≥ 50 mm/h	Hayashi 2012 [13]
3		High Charlson comorbidity index Presence of FC lesions Malignancy	Ito 2012 [14]
4	NB disease	FC disease FC + NB disease Usual interstitial pneumonia Emphysema Other lung disease	Yamakawa 2013 [15]
5	Prior tuberculosis Bronchiectasis Asthma	Male sex Older age Chronic obstructive pulmonary disease Interstitial lung disease Lung cancer HIV infection Cystic fibrosis Bone marrow transplant	Morimoto 2014 [16]
6		Chronic pulmonary aspergillosis Cavitation Emphysema	Zoumot 2014 [17]
7		Male sex Age ≥ 70 years BMI < 18.5 kg/m^2^ Absence of bloody sputum hypoalbuminaemia Erythrocyte sedimentation rate > 40 mm/h	Gochi 2015 [18]
8,9		Male sex Age ≥ 70 years Malignancy, including lung cancer BMI < 18.5 kg/m^2^ Lymphocyte count < 1000/μl FC disease	Kumagai 2017 [19]^a^
10		Male sex Age ≥ 65 years High comorbidity level Positive smear	Andréjak 2010 [20]
11	NTM-PD with multiple species of NTM isolated	Male sex Increasing age Comorbid conditions	Marras 2017 [21]
12		Older age Low BMI Pneumonectomy Remnant cavitary lesions following pulmonary resection	Asakura 2017 [22]^a^
13,14		Lung cancer	Novosad 2017 [23]^a^
15		Increasing age Male sex Involvement of > 1 lung zone Low initial body weight	Research Committee of the British Thoracic Society 2002 [24]
16,17	Adding clarithromycin vs. ciprofloxacin to rifampicin and ethambutol therapy regimen		Jenkins 2008 [25]

*BMI* body mass index, *FC* fibrocavitary disease, *HIV* human immunodeficiency virus, *NB* nodular bronchiectatic disease, *NTM* nontuberculous mycobacteria, *NTM-PD* nontuberculous mycobacterial pulmonary disease

^a^Factors found to be significant by multivariate analysis are listed

Two studies examined the relationship between nodular/bronchiectatic and fibrocavitary MAC lung disease and MAC-related mortality [13, 18]. Both found that patients with fibrocavitary disease had increased five-year MAC-related mortality compared with patients with nodular/bronchiectatic disease (Fig. 3b). One study also analysed the relationship between radiologic types of MAC lung disease and all-cause five-year mortality [13]. This demonstrated that patients with fibrocavitary disease have a substantially greater risk of death compared with nodular disease (Fig. 3c).

### Effect of study region on five-year mortality

We performed a sensitivity analysis using the geographic region in which the selected studies were conducted (Additional file 1: Table S1). The analysis demonstrated that patients in Asian studies tended to have a lower five-year mortality (19, 95% CI 14–23%) compared with Europe (35, 95% CI 27–43%) and North America (33, 95% CI 32–35%).

## Discussion

The studies identified in this systematic review show that, in general, patients with MAC lung disease are at a high risk of death following their diagnosis, with a pooled estimate of five-year all-cause mortality of 27%. In line with previous reports [9], we found there to be considerable heterogeneity between studies, with an I^2^ value of 96% and Q-statistic of 365.1.

Several publications have demonstrated the incremental impact of NTM infection on patient mortality. Adjemian and colleagues found that US patients aged over 65 with NTM-PD within a nationally-representative sample were 40% more likely to die during the study period (1997–2007) than patients without NTM-PD [26]. Recent work from Ontario, Canada, also reported an increased mortality in patients with MAC lung disease compared with a matched control group (HR = 1.57, 95% CI 1.48–1.66, *P* < 0.0001) [21]. Here, the mortality was 33.3% in cases versus 21.5% in controls. Diel et al. identified an even greater mortality risk (HR 3.64, 95% CI 2.28–5.77) and a mortality after 39 months follow-up of 22.4% for NTM-PD patients versus 6.0% for control patients [27]. These studies indicate that NTM-PD increases mortality risk at a population level, independent of underlying comorbidities.

Although predictors of mortality varied between studies, some common features were observed. A worse prognosis was noted with male sex, comorbidities (e.g. coexisting lung disease) and the presence of fibrocavitary disease. The latter was found to be associated with increased MAC-related mortality rate in two studies [13, 18], and in one, all-cause mortality [13]. This is in line with results from Fleshner and colleagues who identified fibrocavitary disease as a predictor of mortality in NTM-PD after controlling for possible confounders (adjusted hazard ratio [aHR] 3.3, 95% CI 1.3–8.3) [28]. Fleshner and colleagues also documented pulmonary hypertension as a risk factor for mortality (aHR 2.1, 95% CI 0.9–5.1), although this was not significant following adjustment for fibrocavitary disease; importantly, individual NTM species were not significantly associated with mortality, suggesting similar risks for each NTM species identified in the study [28].

Relatively few studies have explored differences in mortality between cases with confirmed ATS/IDSA disease criteria against those with NTM isolation only. From our list of identified studies, Marras and colleagues found that mortality rates were higher among patients from Ontario who fulfilled the ATS/IDSA disease criteria compared with those who had NTM isolation only (HR = 1.16, 95% CI 1.09–1.24) [21]. Similarly, five-year age-adjusted mortality rates were slightly higher for patients meeting (28.7/1000) versus not meeting (23.4/1000) ATS/IDSA criteria, respectively, in the report by Novosad identified in our analysis [23]. Andréjak and colleagues noted a similar prognosis in Danish patients with confirmed NTM-PD (57% of whom had MAC isolation) compared with those with NTM isolation only (HR 1.15, 95% CI 0.90–1.51) [20]. Thus, MAC lung disease fulfilling ATS/IDSA criteria is associated with a worse outcome. However, all patients with disease considered bad enough to be recorded by investigators, and hence included in studies, are at some increased risk of death.

Furthermore, whereas all-cause mortality is an objective measure, the proportion of deaths attributed to MAC lung infection depends largely on how clinicians determine the cause of death. This may be unclear, particularly in long-term follow up studies where underlying comorbidities may progress; it a pertinent issue in MAC lung disease as many patients are elderly and often have a number of comorbidities [2, 29]. Thus, the impact of MAC lung disease on mortality at a population level is more appropriately reflected in studies using matched control groups. As shown above, the three studies where MAC lung disease cases were matched with appropriate controls consistently showed an increased risk of mortality for patients with NTM-PD or MAC-PD [21, 26, 27].

Our sensitivity analysis identified a lower mortality rate in Asian studies, particularly those from Japan. A similar trend has previously been observed [30]. This may be driven, in part, by the relatively high proportion of nodular/bronchiectatic disease in Japanese studies [15], which most reports suggest has a better outcome.

The present study has several limitations. We were keen to include a range of studies reflecting the published literature and so did not use a complex set of stringent-pre-specified criteria. Thus, our analysis is influenced by the design of the selected studies. Specifically, only two prospective studies (including three data sets) are included in our analysis [24, 25]. This is challenging for the field as a whole, and further prospective studies of mortality in MAC-PD patients, which could support identification of additional prognostic factors, are warranted.

Furthermore, we could not account for the potential effects of patient immunosuppression (e.g. HIV) or heterogeneity of treatment regimens between studies as the selected studies did not report outcomes for different subgroups. The studies we have identified cover a wide time period (1973–2017) and thus may be influenced by potential variability in NTM diagnosis and treatment over the 44 year period.

A previous meta-analysis of treatment success rates in patients with MAC-PD and MAC-non-PD (the latter including disseminated disease) also found considerable treatment outcome heterogeneity for patients with MAC (I^2^ > 70%, *p* < 0.05 for all treatment outcomes) [9]. The authors suggested that this may be due to inconsistency among treatment protocols and in the reporting of key patient and study characteristics [9], preventing identification of clear factors related to treatment success. However, unlike our study, the authors did not distinguish between MAC-PD and MAC-non-PD.

It is important to note that, as many reported studies are frequently based on population-level data (for example, [21]), they can contain limited clinical information. This inevitably means that one must be careful to not over-interpret their findings.

Most of our selected studies do not explicitly identify patients with macrolide-resistant pulmonary disease. This is a concern, as recent work from Korea reported a five-year mortality of 47.1% (95% CI 24.0–70.1) in patients with macrolide-resistant MAC [31]. This is much higher than the pooled estimate from our analysis indicating that macrolide resistance increases mortality risk, and should be specifically identified in future studies.

## Conclusions

In conclusion, our structured literature review has identified 17 data sets reporting five-year mortality in patients with NTM-PD caused by MAC. Most (i.e. ten) document a five-year mortality rate greater than 25% [12, 14, 15, 20, 21, 23–25], indicating a poor prognosis for patients with MAC lung disease and a need for more effective management of the condition. Substantial heterogeneity in study characteristics was found, with male sex, presence of cavitary disease and high comorbidity levels predicting worse survival outcomes. Further prospective studies using appropriately matched controls may contribute to a better understanding of long-term survival in MAC-related pulmonary disease.

## Additional file

Additional file 1: Search strategies. (DOCX 17 kb)

## Abbreviations

ATS/IDSA: American Thoracic Society/Infectious Diseases Society of America
BTS: The Research Committee of the British Thoracic Society
CF: Cystic fibrosis
CI: Confidence interval
COPD: Chronic obstructive pulmonary disease
MAC: *Mycobacterium avium* complex
NTM: Nontuberculous mycobacterium
NTM-PD: Nontuberculous mycobacteria pulmonary disease
PRISMA: Preferred Reporting Items for Systematic Reviews and Meta-Analyses
