# Supplementary material for: High mortality in patients with Mycobacterium avium complex lung disease: a systematic review
Source: BMC Infect Dis. 2018 May 3;18:206. doi: 10.1186/s12879-018-3113-x (PMC5934808; doi:10.1186/s12879-018-3113-x)
Supplement: Supplementary file 1 — Search strategies. (DOCX 17 kb) [file 12879_2018_3113_MOESM1_ESM.docx]

**Additional file 1 – search strategies.**

Database searches were carried out in Medline^®^ and Embase^®^ using PubMed^®^ and ProQuest Dialog™ search tools, respectively.

Search terms used in PubMed^®^ were: Atypical Mycobacterium Infections; Atypical Mycobacterium Infection; Battey Bacillus; Disseminated Atypical Mycobacteriosis; death; deaths; death rate; death rates; Disseminated Atypical Mycobacterioses; Disseminated Atypical Mycobacteriosis; Familial Atypical Mycobacterioses; Familial Atypical Mycobacteriosis; Familial Disseminated Atypical Mycobacteriosis; Familial Disseminated Atypical Mycobacterioses; fatality; fatal outcome; fatal outcomes; Mycobacterium avium Complex; MAIC; mortality; mortalities; Mycobacterium avium‑intracellulare Complex; Mycobacterium avium-intracellulare; Mycobacterium intracellulare; Mycobacterium avium-intracellulare Infection; Mycobacterium avium‑intracellulare Infection; Mycobacterium avium-intracellulare Infections; Mycobacterium avium intracellulare Infection; Mycobacterium avium intracellulare Infections; Mycobacterium Avium Complex lung disease; Mycobacterium infections; Mycobacterium intracellulare; Nontuberculous; nontuberculous mycobacterial; Nontuberculous Mycobacterium Infection; Nontuberculous Mycobacterium Infections; Non-tuberculous Mycobacterium Infection; Non-tuberculous Mycobacterium Infections; survival; survival analysis; survival rate; survival rates; survivorship. These searches were combined and any case reports excluded.

The Embase^®^ search used the following terms: Atypical Mycobacterium Infection; Atypical Mycobacterium Infections; Battey Bacillus; death; deaths; death rate; death rates; Disseminated Atypical Mycobacterioses; Disseminated Atypical Mycobacteriosis; Familial Atypical Mycobacterioses; Familial Atypical Mycobacteriosis; fatality; fatalities; fatality rate; fatality rates; fatal outcome, fatal outcomes; mortality; mortalities; M avium-intracellulare; MAIC; Mycobacterium avium complex; Mycobacterium Avium Complex lung disease; Mycobacterium avium‑intracellulare Complex; Mycobacterium avium intracellulare Infection; Mycobacterium avium-intracellulare Infection; Mycobacterium avium-intracellulare; Mycobacterium infections; Mycobacterium avium-intracellulare Infections; Mycobacterium intracellulare; Nontuberculous; Nontuberculous mycobacteria; nontuberculous mycobacterial; Nontuberculous Mycobacterium infection; Nontuberculous Mycobacterium infections; Non-tuberculous mycobacterium infection; Non‑tuberculous mycobacterium infection; survival; survival analysis; survival rate; survival rates; survival time; survivorship. These search terms were combined, and case studies and animal studies were excluded. Excluded document types were conference abstracts, newspaper articles, notes, congresses, news, biography, conference reviews, errata and lectures.

Table S1: Summary of five-year mortality in studies by region

| **Region** | **Number of  data sets** | **Mean five-year mortality (%) (95% CI)** | **I^2^** | **Q-statistic** | **Degrees of freedom** |
| --- | --- | --- | --- | --- | --- |
| Europe | 5 | 35% (27–43%) | 62% | 10.55 | 4 |
| North America | 4 | 33% (32–35%) | 0% | 0.98 | 3 |
| Asia | 8 | 19% (14–23%) | 87% | 54.82 | 7 |

CI, confidence interval.
